# The Efficacy of Fecal Microbiota Transplantation in Experimental Autoimmune Encephalomyelitis: Transcriptome and Gut Microbiota Profiling

**DOI:** 10.1155/2021/4400428

**Published:** 2021-12-13

**Authors:** Sanwang Wang, Hongliang Chen, Xin Wen, Jingjing Mu, Mingyue Sun, Xiaowen Song, Bin Liu, Jinbo Chen, Xueli Fan

**Affiliations:** ^1^Department of Psychiatry, Binzhou Medical University Hospital, Binzhou, China; ^2^Department of Neurology, Binzhou Medical University Hospital, Binzhou, China; ^3^Department of Gastroenterology, The Affiliated Huai'an No. 1 People's Hospital of Nanjing Medical University, Huai'an, China; ^4^Institute for Metabolic & Neuropsychiatric Disorders, Binzhou Medical University Hospital, Binzhou, China

## Abstract

**Objective:**

To study the protective effect of fecal microbiota transplantation (FMT) on experimental autoimmune encephalomyelitis (EAE) and reveal its potential intestinal microflora-dependent mechanism through analyses of the intestinal microbiota and spinal cord transcriptome in mice.

**Method:**

We measured the severity of disease by clinical EAE scores and H&E staining. Gut microbiota alteration in the gut and differentially expressed genes (DEGs) in the spinal cord were analyzed through 16S rRNA and transcriptome sequencing. Finally, we analyzed associations between the relative abundance of intestinal microbiota constituents and DEGs.

**Results:**

We observed that clinical EAE scores were lower in the EAE+FMT group than in the EAE group. Meanwhile, mice in the EAE+FMT group also had a lower number of infiltrating cells. The results of 16S rRNA sequence analysis showed that FMT increased the relative abundance of *Firmicutes* and *Proteobacteria* and reduced the abundance of *Bacteroides* and *Actinobacteria*. Meanwhile, FMT could modulate gut microbiota balance, especially via increasing the relative abundance of *g_Adlercreutzia*, *g_Sutterella*, *g_Prevotella_9*, and *g_Tyzzerella_3* and decreasing the relative abundance of *g_Turicibacter*. Next, we analyzed the transcriptome of mouse spinal cord tissue and found that 1476 genes were differentially expressed between the EAE and FMT groups. The analysis of these genes showed that FMT mainly participated in the inflammatory response. Correlation analysis between gut microbes and transcriptome revealed that the relative abundance of *Adlercreutzia* was correlated with the expression of inflammation-related genes negatively, including Casp6, IL1RL2 (IL-36R), IL-17RA, TNF, CCL3, CCR5, and CCL8, and correlated with the expression of neuroprotection-related genes positively, including Snap25, Edil3, Nrn1, Cpeb3, and Gpr37.

**Conclusion:**

Altogether, FMT may selectively regulate gene expression to improve inflammation and maintain the stability of the intestinal environment in a gut microbiota-dependent manner.

## 1. Introduction

Multiple sclerosis (MS) is a chronic inflammatory disease characterized by astroglial injury, axonal loss, demyelination, and inflammation in the brain and spinal cord [1]. The pathogenesis of MS is still unclear, but the general hypothesis is that autoimmunity, viral infection (e.g., Epstein-Barr virus (EBV)), genetic tendency, environmental factors, and individual susceptibility factors play a comprehensive role in the etiology of MS [2, 3]. Moreover, dysbiosis of the commensal gut microbiota is commonly observed in MS patients and might play a pathological role in the inception and progression of this disease [4]. Studies have linked gut dysbiosis to inflammatory bowel disease, local and systemic inflammation, hypertension, type 2 diabetes, and MS [5, 6]. Therefore, modulating the microbiota to correct ecological imbalance may be a new practice to treat MS.

FMT is the transplantation of a fecal microbiota suspension from a healthy donor to a recipient, with the aim of treating or preventing disease via manipulation of the microbiome [7]. Some studies have shown that FMT may be a promising therapy option for nervous system diseases, including MS [8, 9]. It is likely that some elements of donor's healthy gut microbiota can induce rapid production of anti-inflammatory mediators that may counteract proinflammatory cytokines [10]. Notably, fecal transplants from MS patients into germ-free mice have been found to result in more serious symptoms of experimental autoimmune encephalomyelitis (EAE) and reduce the proportions of IL-10^+^ Tregs [11]. Interestingly, gavage with the human gut-derived commensal strain *Prevotella histicola* has been found to bring about a dropped incidence of disease in MS mouse model. The mechanism may be a rebalance between proinflammatory response (including Th1 and 17 cells) and anti-inflammatory response (including Treg cells) [12]. These findings suggest that FMT may exert a therapeutic effect on MS patients by reshaping the intestinal flora and attenuating inflammatory responses.

In this study, we used an EAE mouse model to help develop new therapies for MS and to identify the role of intestinal microflora in coordinating the possible mechanism of FMT on MS. Subsequently, we investigated the relationship between spinal cord transcriptome and intestinal microbiota in the context of inflammation and analyzed the therapeutic effect of FMT on EAE mice. Our discoveries will provide unique insights into the mechanism of intestinal microbiome therapy for MS.

## 2. Materials and Methods

### 2.1. Animals

Female C57BL/6 mice (specific pathogen-free grade), 6-8 weeks of age and weighing 18-20 g (Ji'nan Pengyue Laboratory Animal Breeding Co., Ltd., Jinan, China), were used in the study. These mice were randomly divided into control, EAE, and EAE+FMT groups according to these experimental designs of Wang et al. and Wen et al [13, 14]. All groups of mice were raised under standard humidity, temperature, and a normal diet from day 0 to day 19. All animal studies were conducted in compliance with the National Institutes of Health *Guide for the Care and Use of Laboratory Animals* and carried out according to protocols approved by the Institutional Animal Care and Use Committee of Binzhou Medical University Hospital.

### 2.2. Induction of EAE and Assessment of Clinical Signs

EAE was induced as mentioned previously [15]. Concisely, C57BL/6 mice were injected with 200 *μ*g (MOG) 35-55 peptide (GenScript, New Jersey, USA) emulsified in 100 *μ*g of complete Freund's adjuvant (CFA, Sigma-Aldrich, Missouri, USA) and an additional 400 *μ*g heat-inactivated mycobacterium tuberculosis (Difco, Michigan, USA) by subcutaneous injection. In addition, 300 ng of pertussis toxin (PTX, Merck Millipore, Massachusetts, USA) was injected intraperitoneally on the day of immunization and 48 hours later. Clinical signs were observed and recorded daily by 2 researchers as follows: 0, normal; 1, paralysis or staggering of the tail; 2, mild paralysis of two hind limbs or severe paralysis of one hind limb; 3, severe paralysis of both hind limbs; 4, two hind limbs were severely paralyzed and the forelimbs were affected; 5, moribund or death; and 0.5, intermediate clinical sign (Table 1).

### 2.3. Preparation and Processing of Donor Microbiota for FMT

Donor fecal microbiota was acquired from healthy mice of the same strain as the recipient. Fresh fecal pellets were homogenized in sterile normal saline and then centrifuged at 1500 rpm for 5 min at 4°C. The sediment was collected and resuspended in sterile normal saline at 0.125 g/mL. The EAE+FMT group was given 200 *μ*l per mouse fresh fecal suspension via oral gavage, whereas for the EAE group, fecal suspension was replaced with sterile saline. Transplantation was from the 10th day of the experiment, once every two days, until the end of the experiment.

### 2.4. Histopathology

On day 19, we took out the spinal cords of mice after being perfused by intracardiac infusion with 4% paraformaldehyde. Then, the spinal cords were 4% neutral-buffered formalin fixed, embedded in paraffin wax, sectioned at 4-6 *μ*m, and stained with hematoxylin-eosin (HE) for pathological examination. The stained sections were evaluated by 2 researchers scored as 0, no cell infiltration; 1, the infiltration of inflammatory cells appeared in the spinal membrane; 2, inflammatory cell aggregation and infiltration in 1–2 blood vessels; 3, inflammatory cell aggregation in 3–4 blood vessels and/or a large range of 1 parenchymal involvement; and 4, a large number of cell infiltration involving over 20% of the region (Table 2).

### 2.5. 16S rRNA Sequencing

Fecal samples of 3 groups were collected on the last day (day 19) of experiment. 18 randomly selected stool samples (*n* = 6 each group) were stored until extraction at −80°. The V3-V4 hypervariable regions of the bacterial 16S rRNA gene were amplified with primer as follows: 338F (5′-ACTCCTACGGGAGGCAGCAG-3′) and 806R (5′-GGACTACHVGGGTWTCTAA T-3′) by an ABI GeneAmp® 9700 PCR thermocycler (ABI, CA, USA). All PCR products were extracted from 2% agarose gel and purified using the AxyPrep DNA Gel Extraction Kit (Axygen Biosciences, Union City, CA, USA). Purified amplicons were sequenced on the Illumina MiSeq PE300 platform (Illumina, San Diego, USA). The raw reads of 16S rRNA sequencing were deposited into the NCBI Sequence Read Archive (SRA) database (Accession Number: SRP332417).

### 2.6. Transcriptome Analysis

Then, we collected mouse spinal cord samples on day 19 from the EAE and EAE+FMT groups for sequencing (*n* = 3 each group). RNA degradation and contamination were monitored on 1% agarose gels and sequenced on the Illumina Hiseq 4000 platform. Differential expression analysis was performed using the DESeq R package (1.10.1) according to the manufacturer's protocol. Next, to explore the potential function of the DEGs, we used the GOseq R packages and KOBAS software to test the statistical enrichment of DEGs in Gene Ontology (GO) functional annotation and Kyoto Encyclopedia of Genes and Genomes (KEGG) pathway. The raw reads of transcriptome sequencing were deposited into the NCBI Sequence Read Archive (SRA) database (Accession Number: SRP332501).

### 2.7. Correlation Analysis between Gut Microbiota and Transcriptome

We used Metastats software to confirm whether there was any difference in the relative abundance of microbiota among the samples (*P* ≤ 0.05). Then, we use DEGseq to do transcriptome difference analysis (the threshold is *P*adj < 0.05 and ∣log_2_FC | >1). Finally, R psych software package was used to make the Spearman association between the transcriptome and the intestinal microflora. ∣*R* | >0.8 and *P* < 0.05 (strong correlation) are screened for mapping [14].

### 2.8. Statistical Analysis

Analysis of statistical significance was performed using GraphPad Prism version 8.0 (GraphPad Software Inc., San Diego, USA) and SPSS 26.0 (IBM Deutschland, Ehningen, Germany). Student's *t*-test was used to compare values between two groups. Values of *P* < 0.05 were considered as statistically significant.

## 3. Results

### 3.1. FMT Ameliorated the Severity of EAE Disease

**Figure 1:**
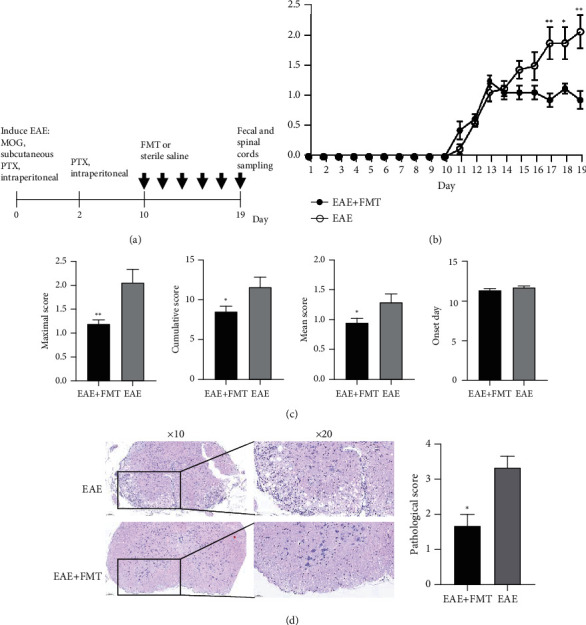
FMT has a therapeutic effect on EAE. (a) Experimental design of FMT treatment in an EAE model. (b) Daily changes in clinical scores between the two groups during the disease process (mean ± SEM; *n* = 8 each; ^∗^*P* < 0.05 and ^∗∗^*P* < 0.01). (c) The mean ± SEM of the maximal scores, cumulative scores, mean scores, and onset day of the EAE group and EAE+FMT group. The cumulative, maximum, and mean EAE scores of the EAE+FMT group were significantly lower than those of the EAE group (mean ± SEM; *n* = 8 each; ^∗^*P* < 0.05 and ^∗∗^*P* < 0.01). (d) Serial sections of spinal cord tissues were stained with H&E (mean ± SEM; *n* = 3 each; ^∗^*P* < 0.05 and ^∗∗^*P* < 0.01) (original magnifications: 10x (left) and 20x (right)). Pathological scores of each group were determined. EAE: experimental autoimmune encephalomyelitis; EAE+FMT: fecal microbiota transplantation.

To determine the mechanisms through which FMT attenuates EAE, we undertook studies as described in Figure 1. The control group included 8 healthy mice. Mice in the EAE group (*n* = 8) were gavaged with normal saline starting from day 10, while the EAE+FMT group (*n* = 8) was gavaged with fecal microbiota separated from healthy donor at the same time (Figure 1(a)). Compared with that of the EAE group, the EAE+FMT group obviously alleviated disease severity according to clinical EAE scores (*P* < 0.01; Figures 1(b)–1(c)). This phenomenon suggested that this kind of treatment effectively improved the symptoms in the EAE model. In order to further evaluate the therapeutic effect of FMT, H&E staining was used to evaluate pathological features. Histological results from the spinal cords on day 19 revealed that EAE mice (*n* = 3) had a higher number of infiltrating cells when compared to EAE+FMT mice (*n* = 3) (Figure 1(d)). Taken together, these results suggested that FMT alleviated the progression of EAE disease and inflammatory response in mice.

### 3.2. FMT Changed the Diversity and Composition of the Gut Microbiota during EAE Induction

**Figure 2:**
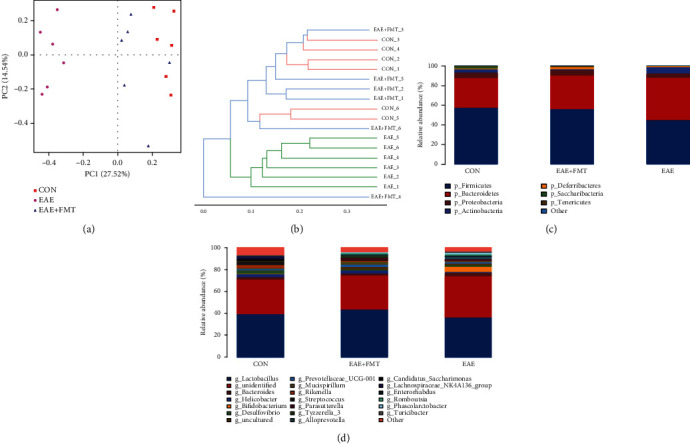
FMT significantly altered the gut microbiota structure and composition during EAE disease. (a) Multiple-sample principal component analysis (PCA). (b) System clustering tree of the gut microbiota based on unweighted UniFrac metrics indicating the beta diversity of the gut microbiota in each group. (c, d) Stacked bar graphs of the relative abundance at the phylum and genus levels (*n* = 6 each). CON: control; EAE: experimental autoimmune encephalomyelitis; EAE+FMT: fecal microbiota transplantation.

To investigate the role of FMT treatment on microbiota, sequencing of 16S rRNA gene was measured (*n* = 6 each group). Fecal samples of these 3 groups were collected on day 19. Principal component analysis (PCA) of Bray-Curtis distance matrices was carried out for beta diversity determination among different groups. As shown in Figure 2(a), evident separation of the gut microbiota was observed on the two-dimensional PCA plots among different groups. In the EAE group, the gut microbiota shifted significantly along the direction of PC1 and PC2, deviating from the baseline structure. A hierarchical clustering tree (Figure 2(b)) showed significant differences between each group. FMT-treated samples were clustered separately from those of the EAE group but closely to those of the control group, reflecting that FMT could alleviate the changes in the gut microbiota during EAE induction. The results showed a significant difference in microbial beta diversity between the three groups, indicating that the distribution of species in EAE was uneven, which could be rebalanced by FMT.

**Figure 3:**
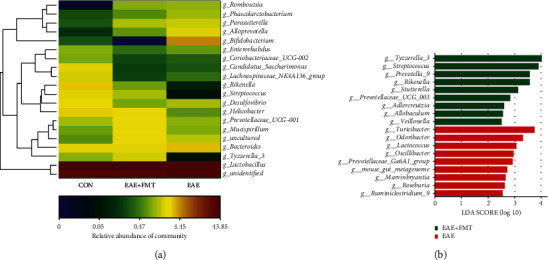
Differences in dominant microorganisms among groups based on genus abundance. (a) Heat map of the most differentially abundant features at the genus level. Relative abundance is indicated by a color gradient from blue to red, with blue representing low abundance and red representing high abundance. (b) LEfSe analysis of the groups (*n* = 6 each). CON: control; EAE: experimental autoimmune encephalomyelitis; EAE+FMT: fecal microbiota transplantation.

Histograms demonstrate the gut microbiota community structure and depict the differences in the relative abundance of major gut microbiota at the phylum and genus levels. In terms of bacterial composition at the phylum level, EAE decreased the levels of *Firmicutes* and *Proteobacteria* (21.45% and 24.20%, respectively) and increased the levels of *Bacteroidetes* and *Actinobacteria* (43.83% and 93.69%, respectively) compared to those in the control group (Figure 2(c)). However, following FMT treatment, the proportions of *Firmicutes*, *Proteobacteria*, *Bacteroidetes*, and *Actinobacteria* returned to levels comparable to those in the control group. After FMT treatment, the relative abundance of some specific genera changed (Figure 2(d)).  Figure 3(a) shows a hierarchically clustered heat map of the fecal microbiota composition at the genus level. Results of heat map and LEfSe analysis showed that at the genus level, some genus (*g_Tyzzerella_3*, *g_Prevotella_9*, *g_Sutterella*, *g_Rikenella*, and *g_Adlercreutzia*) were enriched in the FMT group, and others (*g_Turicibacter*, *g_Prevotellaceae_Ga6A1_group*, *g_Parasutterella*, *g_Marvinbryantia*, and *g_Alloprevotella*) were enriched in the EAE group (Figure 3(b)). Altogether, the above results indicated that FMT could rebalance the intestinal flora during EAE induction.

### 3.3. FMT Regulated the Inflammatory Response in the Spinal Cord

**Figure 4:**
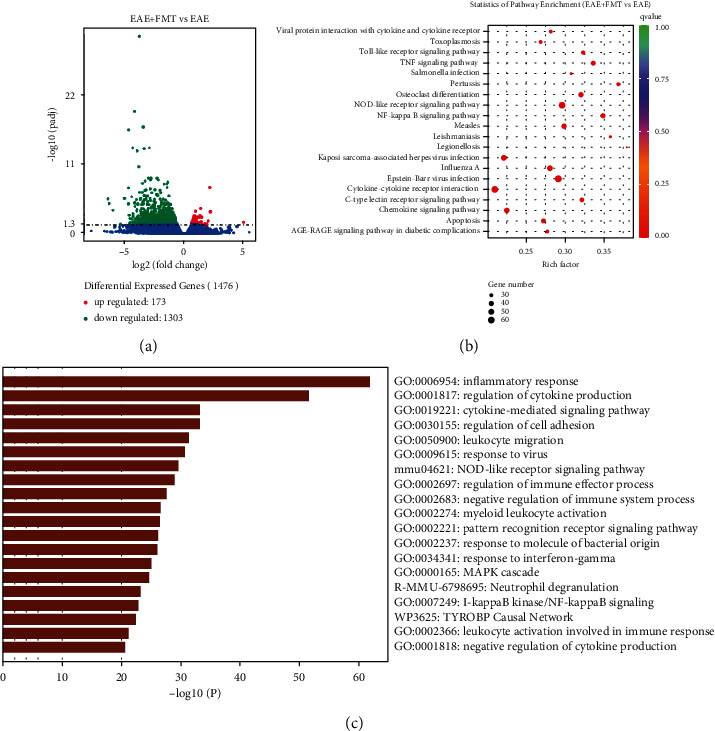
Functional analysis of the effects of FMT on the spinal cord transcriptome in EAE mice. (a) Volcano plot of significant DEGs regulated between the EAE and FMT groups. (b) KEGG pathway analysis showing the top 30 signaling pathways affected by FMT. (c) DEGs analyzed in Metascape.

Subsequently, to investigate further mechanism of FMT, we employed spinal cord RNA sequencing from the EAE (*n* = 3) and EAE+FMT (*n* = 3) groups. The volcano plot in Figure 4(a) shows the DEGs between the EAE and EAE+FMT groups. A total of 1476 DEGs showed changes in expression between the two groups, among which 173 genes were upregulated and 1303 genes were downregulated (Figure 4(a)). To gain a better understanding of how these DEGs may delay the onset of EAE and decrease the disease severity of EAE, KEGG and GO enrichment analyses were performed.  Figure 4(b) shows the results of the KEGG pathway analysis of DEGs. The main enriched KEGG pathways were Epstein-Barr virus infection, NOD-like receptor signaling pathway, NF-*κ*B signaling pathway, and TNF signaling pathway. The top 10 most significant KEGG pathway terms are shown in Table 3. The differential genes of inflammation and neuroprotection between the EAE group and the EAE+FMT group are listed in Table 4. GO analysis indicated that 1476 DEGs mainly engaged in the inflammatory response and regulation of cytokine production (Figure 4(c)). The enrichment analyses indicated that FMT not only might play a vital role in rebalancing the microbiota but also is important in the inflammatory response.

### 3.4. Interactions between Inflammation/Neuroprotection-Related Genes and the Gut Microbiota

**Figure 5:**
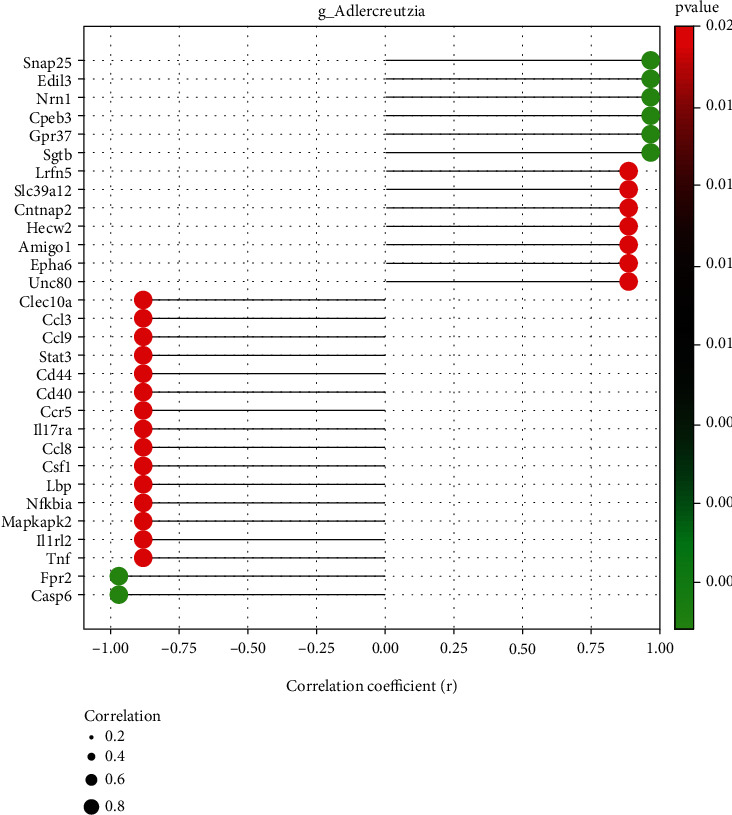
Correlations between relative genus abundance and the transcriptome. (a) A lollipop shape displayed microbe-gene correlations (∣*R* | >0.8, *P* < 0.05). Positive values indicate positive correlations, and negative values indicate negative correlations.

According to aforementioned findings, we attempted to identify the potential signal pathways mediated by the immune axis of the microbiota. Correlation analysis illustrated that *Adlercreutzia* was the most abundant genus associated with the DEGs (Figure 5). The DEGs associated with inflammation, such as Casp6, IL1RL2 (IL-36R), IL-17RA, TNF, CCL3, CCR5, and CCL8, were negatively correlated with the relative abundance of *Adlercreutzia* (∣*R* | >0.8 and *P* < 0.05). The DEGs associated with synaptoprotective effects and decreased demyelination, such as Snap25, Edil3, Nrn1, Cpeb3, and Gpr37, were correlated with the relative abundance of *Adlercreutzia* positively (∣*R* | >0.8 and *P* < 0.05). These results indicate a potential *Adlercreutzia*-mediated immune regulation mechanism for FMT treatment of EAE.

## 4. Discussion

MS is the most prevalent chronic inflammatory disease of the central nervous system, affecting millions of individuals especially young adults worldwide [16]. However, the underlying cause of MS remains opaque and it is currently incurable. Recently, increasing clinical and preclinical studies indicate that gut microbiome is a possible key susceptibility factor for MS and altered microbial composition plays a great role in the pathophysiology of MS [17, 18]. In our study, we found that the clinical score and pathological changes of EAE mice were alleviated after FMT. Similar to our study, a previous study found that FMT has a certain therapeutic effect on the EAE model [19].

Afterwards, we measured sequencing of 16S rRNA gene of fecal samples. Compared with the EAE group, the EAE+FMT group displayed and altered the beta diversity of the intestinal microbial community, which is close to the control group. Meanwhile, we found the relative abundance of conditional pathogens (such as *g_Turicibacter* and *g_Parasutterella*) increased, while the relative abundance of protective bacteria (such as *g_Adlercreutzia*, *g_Prevotella_9*, and *g_Sutterella*) decreased in EAE, which is consistent with the trend of the fecal microbiota in patients with MS [4, 20, 21]. After FMT, *g_Adlercreutzia*, *g_Prevotella_9*, and *g_Sutterella* became obviously enriched, compared with the EAE group. Hence, FMT may reverse the EAE-associated microbiota constituents. Some studies have reported that the *Turicibacter* genus was relevant to host inflammation and leads to brain vascular dysfunction [22], via producing proinflammatory factors, such as IL-6, IL-1*β*, TNF-*α*, and IFN-*γ* [23]. *Parasutterella*, a genus of Betaproteobacteria, was correlated with TNF-*α*, IL-6, and IL-1*β* levels positively [24, 25]. However, a few data have demonstrated that *Parasutterella* actively participates in metabolic functionality, particularly the deconjugation process of taurine-conjugated bile acids [26], while *Turicibacter* had a beneficial role in host serotonin metabolism [27]. It has been shown that *Adlercreutzia* has an inverse correlation with cardiovascular functions [28], diabetes [29], and gestational diabetes mellitus (GDM) [30], which regulates phytoestrogenic activity, produces short-chain fatty acids (SCFAs), and has neuroprotective effects. In line with our study, increasing studies on human MS have indicated that individual or combined strains of *Prevotella* may have anti-inflammatory and neuroprotective effects [20], through enhancing the production of protective SCFAs [31], increasing the production of phytoestrogens [32], and mediating Th17-related immune responses [33]. Jangi et al. observed increases in the abundance of the genus *Sutterella* in MS patients after immunomodulatory therapy [34]. Overall, these results indicate that the ameliorative effect of FMT on EAE may be related to gut microbiota regulation, restoring the gut microbiota to a beneficial mode for organisms.

Next, we further analyzed spinal cord RNA sequencing in the EAE and EAE+FMT groups. We found 1476 DEGs between the groups, among which 173 genes were upregulated and 1303 genes were downregulated. In addition, GO analysis indicated that 1476 DEGs mainly engaged in the inflammatory response and regulation of cytokine production, while the main enriched KEGG pathways were EBV infection, NOD-like receptor signaling pathway, NF-*κ*B signaling pathway, and TNF signaling pathway. EBV, a unique double-stranded DNA *γ*-herpesvirus involved in the process of infecting, activating, clonally expanding, and persisting latently in B lymphocytes for the lifetime of the infected individual, plays a major role in the development of MS [35]. NOD-like receptor protein 3 (NLRP3) enhance inflammation and T cell responses in MS and EAE [36, 37]. Another NOD-like receptor Nlrp12, negatively regulating NF-*κ*B pathway, has been found suppressing inflammation and exerting a protective role in EAE [36]. Moreover, the DEGs between the EAE group and EAE+FMT group showed that FMT downregulated the expression of inflammation-related genes, including Casp6, IL1RL2 (IL-36R), IL-17RA, TNF, CCL3, CCR5, and CCL8, while upregulated the expression of neuroprotection-related genes, including Snap25, Edil3, Nrn1, Cpeb3, and Gpr37. Recent research has suggested that Casp6 mediates innate immunity and inflammasome activation [38] and it is related to axonal degeneration and cognitive impairment in Alzheimer's disease (AD) [39]. It is well known that proinflammatory factors, such as IL-1, IL-17, and TNF, play an important role in the pathogenesis of MS and EAE [40–42]. IL-36*α*, IL-36*β*, and IL-36*γ* are members of the IL-1 family that signal through a common receptor composed of IL-36 receptor (IL-36R) and IL-1R accessory protein (IL-1RAcP) to activate NF-*κ*B and MAPKs and produce proinflammatory molecules such as IL-1a/b, IL-6, and IL-8 [43]. Interleukin-17A (IL-17A) is a founding member of a novel family of inflammatory cytokines [44], and inhibition of IL-17-induced NOTCH1 activation can attenuate EAE [40]. TNF-*α*, an inflammatory mediator, has the ability to weaken the expression of tight junction proteins consequently enhancing the intestinal barrier permeability [45]. Chemokines and chemokine receptors are key mediators for the recruitment and migration of immune cells to inflammatory sites in the central nervous system [46–48], which are increased in EAE models and patients with MS [47, 49, 50]. Our results are in agreement with these findings, as FMT led to reduction in chemokine receptors and their ligands, such as CCL3, CCR5, and CCL8. Edil3 has been shown to act as an endogenous homeostasis factor in the central nervous system, protecting neuroinflammation and demyelination through the IL-17/neutrophil inflammatory axis [51]. Snap25 plays a critical role in synaptic function and is considered an important indicator of neuronal recovery after EAE treatment [52]. Nrn1 is involved in the survival and differentiation of nerve cells, the growth of axons and dendrites, and the formation and maturation of synapses. Overexpression of Nrn1 effectively promotes the regeneration of optic nerve and the recovery of visual function [53]. Cpeb3 and Gpr37f have been shown to affect synaptic plasticity and myelination, respectively [54, 55]. In addition, gpr37 may become a potential drug target for the treatment of demyelinating diseases such as multiple sclerosis.

**Figure 6:**
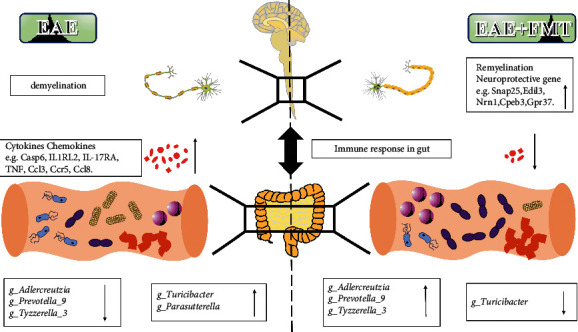
Suggested mechanism by which FMT protects EAE mice from the inflammatory response and demyelination.

Furthermore, we investigated the association between spinal cord transcriptome and the gut microbiota. We found that there was a high negative correlation between *Adlercreutzia* and inflammatory factors, such as Casp6, IL1RL2 (IL-36R), IL-17RA, TNF, CCL3, CCR5, and CCL8. Intestinal microbiota, including *Adlercreutzia*, is known to transform phytoestrogens into bioactive phytoestrogens, such as genistein, equol, and enterolignan [56]. Studies have shown that genistein reducing proinflammatory cytokines (including TNF-*α*, IFN-*γ*, and IL-12p40) can significantly ameliorate EAE [57]. Similarly, 7-O-tetradecanoyl (TDG), a lipophilic genistein analog, suppressed disease when administered subcutaneously 14 days after EAE induction. In these studies, disease amelioration correlated with a decrease in the number of IL-17-producing CD4+ T cells and an increase in the number of FoxP3+ CD4+ T cells in the brain [58]. Moreover, the DEGs associated with synaptoprotective effects and decreasing demyelination, such as Snap25, Edil3, Nrn1, Cpeb3, and Gpr37, were correlated with the relative abundance of *Adlercreutzia* positively. Our study reveals the association between *Adlercreutzia* and the abovementioned neuroprotective genes, which have not been shown in previous studies. These results indicate a potential *Adlercreutzia*-mediated immune regulation mechanism for FMT treatment of EAE (Figure 6).

Gut microbiota and the CNS have bidirectional communication pathways through the microbiota-gut-brain axis [59], which involves many pathways such as the nervous system (including vagus nerve, enteric nervous system, and spinal nerve), the hypothalamic-pituitary-adrenal (HPA) axis, the immune system (including immune cells and cytokines), the endocrine system (including gut hormones), the neuroactive pathway (including neurotransmitters and neuroactive metabolites), and microbe and its metabolites (including short-chain fatty acids (SCFA) and key dietary amino acids) [37, 60, 61]. In MS, the bacteria in favor of inducing immunoregulatory cells (including IL-10+ Tregs) is lacking, while the bacteria in favor of inducing proinflammatory response is enriched [62, 63]. Moreover, gut dysbiosis, increased intestinal permeability, microbial translocation, and local and systemic inflammation are associated with the animal models of MS [45]. Balanced microbiome by FMT may convert the immune process towards immunoregulatory response depending on regulatory cells (Treg) and their anti-inflammatory cytokines (IL-10, TGF-*β*, and IL-35) which play a vital role in recovering immune homeostasis and protecting against CNS inflammatory demyelination [45]. Similarly, FMT was supposed to reduce the production of inflammatory cytokines and trigger immune-mediated signal pathways in colitis [64]. What is more, modified gut microbiota by FMT promotes increasing the compromised epithelial integrity via reducing inflammatory mediators as well as the direct production of metabolites (including SCFA) [45]. However, the precise mechanisms of FMT in MS and EAE require further research.

Providers, housing laboratory, mouse strain, diet, and age are important factors that influence the composition of mouse gut microbiome, which should be taken into consideration during the design and the conclusions of experiments in mice [65]. A previous study showed that the gut microbiome of different mouse strains (BALB/c, B6.*V-Lep^ob^*/J, and NOD) is similar to each other on the bacterial relative distribution on higher taxonomic levels [66], while another study indicated mouse strain had remarkable effect on the abundance of Akkermansia and Lactobacillus [65]. Also, a recent study has shown that a probiotic played differential roles on allergic airway inflammation in A/J and C57BL/6 mice and was associated with gut microbiome [67]. Although gut microbiota is stable, it varies both in time and in different strains.

The current study has certain limitations. First, the sequencing sample size was relatively small, so the sample size should be further expanded for in-depth research. Then, our study lacks specific mechanisms of FMT treatment. Next, to evaluate the mechanism of FMT on MS, we lack human studies. At last, the probable differences among diverse species of mice should be taken into consideration. We are planning to transplant fecal microbiota from EAE, other species of mice, or transplant specific gut microbiota like *Adlercreutzia* for further study. Therefore, our further goal is to conduct clinical studies and in vitro cellular experiments to better understand the relationship between the gut microbiota and its metabolites in immune dysregulation and MS pathogenesis.

## 5. Conclusion

Although FMT is a promising treatment for MS, the potential mechanism of its effects on the intestinal microbiota and the CNS still needs further study. Overall, our research provides a proof of concept for the following main ideas. We explained that FMT has a potential macroscopic therapeutic effect on EAE mice, reducing the clinical scores and pathologic outcomes of EAE. This mechanism is partly because FMT regulates the structure of the gut microbiota by increasing the abundance of beneficial bacteria (such as *Adlercreutzia*, *Sutterella*, and *Prevotella_9)* and reducing the abundance of pathogenic bacteria (such as *g_Turicibacter*). This change in the intestinal microflora may be closely related to the relief of neurological dysfunctions in EAE mice. Finally, our study demonstrated that alteration of the gut microbiota can modulate inflammation and demyelination in EAE mice. Our experiment reveals the relationship between the intestinal microbiota and spinal cord tissue transcriptome for the first time, which provides new clues for the treatment of MS.

## Figures and Tables

**Table 1:** Clinical scores in EAE mice.

	Clinical score
Normal	0
For intermediate clinical sign	0.5
Paralysis or staggering of the tail	1
Mild paralysis of two hind limbs or severe paralysis of one hind limb	2
Severe paralysis of both hind limbs	3
Two hind limbs were severely paralyzed and the forelimbs were affected	4
Moribund or death	5

**Table 2:** Pathological scores of the spinal cords in EAE mice.

	Pathological score
No cell infiltration	0
The infiltration of inflammatory cells appeared in the spinal membrane	1
Inflammatory cell aggregation and infiltration in 1–2 blood vessels	2
Inflammatory cell aggregation in 3–4 blood vessels and/or a large range of 1 parenchymal involvement	3
A large number of cell infiltration involving over 20% of the region	4

**Table 3:** KEGG pathways with statistical enrichment of DEGs.

ID	Pathway	Gene number	*P* value
mmu05169	Epstein-Barr virus infection	67	6.18*E* − 09
mmu04621	NOD-like receptor signaling pathway	61	1.76*E* − 08
mmu04064	NF-kappa B signaling pathway	38	3.15*E* − 07
mmu04668	TNF signaling pathway	38	6.55*E* − 07
mmu04380	Osteoclast differentiation	41	6.90*E* − 07
mmu05162	Measles	43	1.74*E* − 06
mmu04625	C-type lectin receptor signaling pathway	36	2.99*E* − 06
mmu05164	Influenza A	46	3.20*E* − 06
mmu05133	Pertussis	28	4.61*E* − 06
mmu04620	Toll-like receptor signaling pathway	32	9.41*E* − 06

**Table 4:** List of differentially expressed genes of EAE+FMT and EAE.

Number	Gene name	Log_2_ fold change	*P* value	*P* adj
1	Casp6	-1.7316	<0.001	0.02359
2	IL1RL2	-3.062	0.00175	0.04262
3	IL-17RA	-1.1551	<0.001	0.02615
4	TNF	-3.6648	<0.001	<0.001
5	CCL3	-3.1642	<0.001	0.00631
6	CCR5	-1.9686	<0.001	0.02097
7	CCL8	-1.8208	<0.001	0.01853
8	Snap25	0.5866	0.00161	0.04058
9	Edil3	0.60373	0.00119	0.03343
10	Nrn1	0.63477	0.00161	0.04058
11	Cpeb3	0.60083	0.00169	0.04164
12	Gpr37	0.57636	0.00207	0.04663

## Data Availability

The raw reads of 16S rRNA sequencing and transcriptome sequencing were deposited into the NCBI Sequence Read Archive (SRA) database (Accession Number: SRP332417 and SRP332501).
